# The genome sequence of the Dotted Border, *Agriopis marginaria* (Fabricius, 1776)

**DOI:** 10.12688/wellcomeopenres.19284.1

**Published:** 2023-03-31

**Authors:** David Lees, Douglas Boyes, Natural History Museum Genome Acquisition Lab, University of Oxford and Wytham Woods Genome Acquisition Lab, Darwin Tree of Life Barcoding collective, Wellcome Sanger Institute Tree of Life programme, Wellcome Sanger Institute Scientific Operations: DNA Pipelines collective, Tree of Life Core Informatics collective, Darwin Tree of Life Consortium

**Affiliations:** 1 Natural History Museum, London, England, UK; 2 UK Centre for Ecology & Hydrology, Wallingford, England, UK

**Keywords:** Agriopis marginaria, Dotted Border, genome sequence, chromosomal, Lepidoptera

## Abstract

We present a genome assembly from an individual male *Agriopis marginaria* (the Dotted Border, Arthropoda; Insecta; Lepidoptera; Geometridae). The genome sequence is 500.9 megabases in span. Most of the assembly is scaffolded into 29 chromosomal pseudomolecules, including the assembled Z sex chromosome. The mitochondrial genome has also been assembled and is 16.9 kilobases in length. Gene annotation of this assembly on Ensembl identified 12,443 protein coding genes.

## Species taxonomy

Eukaryota; Metazoa; Ecdysozoa; Arthropoda; Hexapoda; Insecta; Pterygota; Neoptera; Endopterygota; Lepidoptera; Glossata; Ditrysia; Geometroidea; Geometridae; Ennominae; *Agriopis*; *Agriopis marginaria* (Fabricius, 1776) (NCBI:txid190331).

## Background

The Dotted Border, *Agriopis marginaria*, is a geometrid moth, the female being brachypterous, spider-like, and the male fully winged, moderately large and varying from greyish to brown or even melanic on the forewing, usually with a paler median fascia and distinguished by around seven prominent dark dots along the termen of both wings. The moth emerges in early Spring (usually flying from February to late April in the UK, with a peak in mid-March that has advanced in 30 years; Randle *et al.*, 2019), occasionally from late December.

The Dotted Border is found in a wide variety of wooded and open scrubby habitats. The larvae are polyphagous, feeding on a diversity of deciduous trees and shrubs, especially oaks.

*A. marginaria* is generally common, sometimes abundant and widespread in the western Palaearctic only, from Scandinavia to the circum-Mediterranean; but has relatively few records for eastern Europe and western Russia, Iceland and Northern Africa ( GBIF Secretariat, 2022). In the UK it is also widespread and often common with relatively fewer records towards the north ( NBN Atlas Partnership, 2021). Populations evidence a significant decline in abundance and a moderate decline in distribution since 1970 ( Conrad *et al.*, 2006; Randle *et al.*, 2019).

There was a single DNA barcode cluster on BOLD (8 March 2023), the BIN BOLD:AAC0355, another (BOLD:AES2645) apparently being artefactual (the available 347 bp of a sequence from Czech Republic are of the same haplotype as some members of the other BIN).

*A. marginaria* is a member of the ennomine tribe Boarmiini, and *Agriopis* (Hübner, 1825) fell sister to the genus *Calamodes* (Guenée, 1857) in the study of Murillo-Ramos *et al.* ( Murillo-Ramos *et al.*, 2021), from which it has been estimated to have diverged around 30 Ma and about 9.6 Ma from *A. aurantiaria*. *Agriopis* is currently placed in the tribe Bistonini. The genome sequence will be useful in further phylogenetic studies, and also to understand evolution of various traits like larval polyphagy and adult flightlessness, as well as of the development of melanism (see e.g., Majerus (1998)) in comparison to the Peppered Moth, *Biston betularia* (L.), whose genome is available ( Boyes & Wright, 2022).

## Genome sequence report

**Figure 1.**
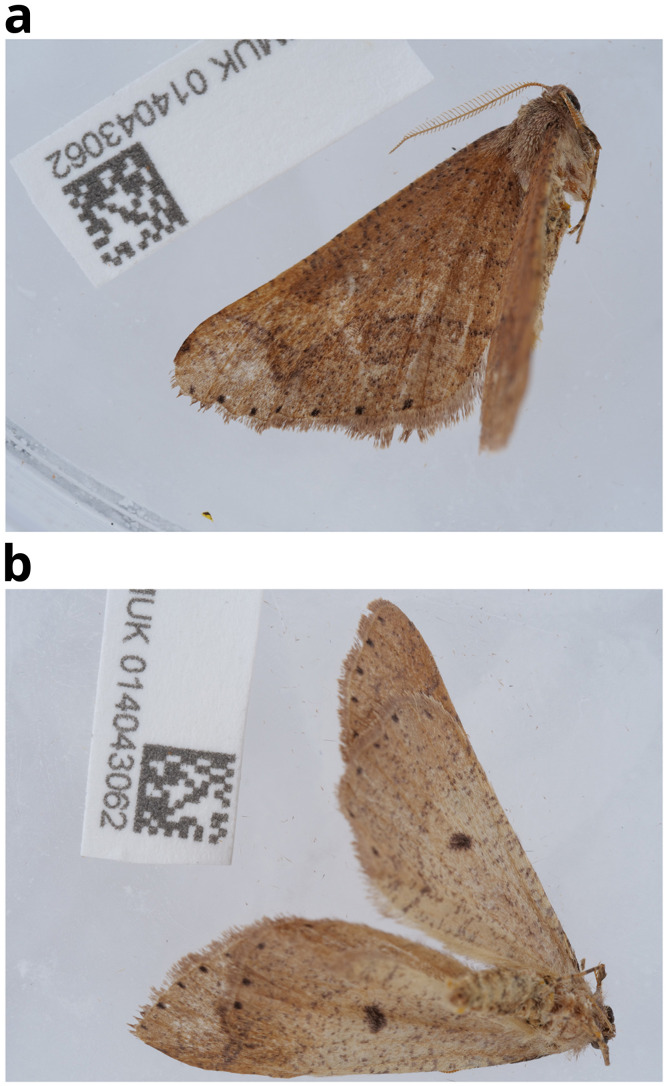
Photograph of the *Agriopis marginaria* (ilAgrMarg1) specimen used for genome sequencing. **a**) Lateral view **b**) Ventral view.

The genome was sequenced from one male *Agriopis marginaria* ( Figure 1) collected from High Wycombe (see Methods). A total of 34-fold coverage in Pacific Biosciences single-molecule HiFi long reads was generated. Primary assembly contigs were scaffolded with chromosome conformation Hi-C data. Manual assembly curation corrected 25 missing or mis-joins and removed 17 haplotypic duplications, reducing the assembly length by 3.12% and the scaffold number by 24.39%, and decreasing the scaffold N50 by 5.06%.

**Figure 2.**
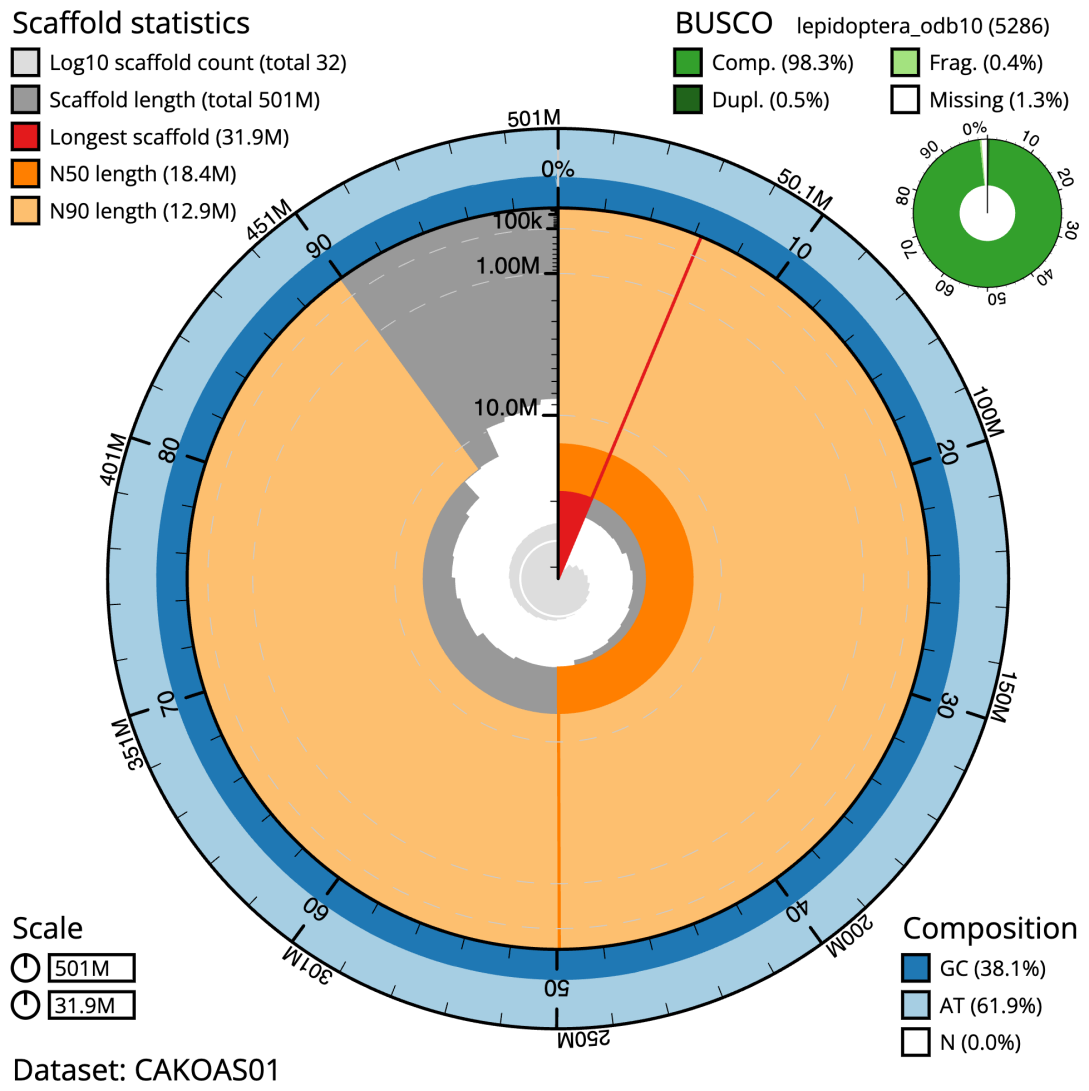
Genome assembly of *Agriopis marginaria*, ilAgrMarg1.1: metrics. The BlobToolKit Snailplot shows N50 metrics and BUSCO gene completeness. The main plot is divided into 1,000 size-ordered bins around the circumference with each bin representing 0.1% of the 500,896,711 bp assembly. The distribution of scaffold lengths is shown in dark grey with the plot radius scaled to the longest scaffold present in the assembly (31,945,455 bp, shown in red). Orange and pale-orange arcs show the N50 and N90 scaffold lengths (18,395,758 and 12,875,431 bp), respectively. The pale grey spiral shows the cumulative scaffold count on a log scale with white scale lines showing successive orders of magnitude. The blue and pale-blue area around the outside of the plot shows the distribution of GC, AT and N percentages in the same bins as the inner plot. A summary of complete, fragmented, duplicated and missing BUSCO genes in the lepidoptera_odb10 set is shown in the top right. An interactive version of this figure is available at https://blobtoolkit.genomehubs.org/view/ilAgrMarg1.1/dataset/CAKOAS01/snail.

The final assembly has a total length of 500.9 Mb in 31 sequence scaffolds with a scaffold N50 of 18.4 Mb ( Table 1). Most (99.99%) of the assembly sequence was assigned to 29 chromosomal-level scaffolds, representing 28 autosomes, and the Z sex chromosome. Chromosome-scale scaffolds confirmed by the Hi-C data are named in order of size ( Figure 2– Figure 5; Table 2). While not fully phased, the assembly deposited is of one haplotype. Contigs corresponding to the second haplotype have also been deposited. The mitochondrial genome was also assembled and can be found as a contig within the multifasta file of the genome submission.

**Table 1.** Genome data for *Agriopis marginaria*, ilAgrMarg1.1.

Project accession data		
Assembly identifier	ilAgrMarg1.1	
Species	*Agriopis marginaria*	
Specimen	ilAgrMarg1	
NCBI taxonomy ID	190331	
BioProject	PRJEB50734	
BioSample ID	SAMEA9359455	
Isolate information	ilAgrMarg1, male: abdomen (genome sequencing); head and thorax (Hi-C) ilAgrMarg2: abdomen (RNA sequencing)	
Assembly metrics *		*Benchmark*
Consensus quality (QV)	64.6	*≥ 50*
*k*-mer completeness	100%	*≥ 95%*
BUSCO **	C:98.3%[S:97.8%,D:0.5%], F:0.4%,M:1.3%,n:5,286	*C ≥ 95%*
Percentage of assembly mapped to chromosomes	99.99%	*≥ 95%*
Sex chromosomes	Z chromosome	*localised homologous pairs*
Organelles	Mitochondrial genome assembled	*complete single alleles*
Raw data accessions		
PacificBiosciences SEQUEL II	ERR8575367	
Hi-C Illumina	ERR8571649	
PolyA RNA-Seq Illumina	ERR10123669	
Genome assembly		
Assembly accession	GCA_932305915.1	
*Accession of alternate haplotype*	GCA_932301435.1	
Span (Mb)	500.9	
Number of contigs	52	
Contig N50 length (Mb)	14.5	
Number of scaffolds	31	
Scaffold N50 length (Mb)	18.4	
Longest scaffold (Mb)	32.0	
Genome annotation		
Number of protein-coding genes	12,443	
Number of non-coding genes	1,975	
Number of gene transcripts	21,487	

^*^Assembly metric benchmarks are adapted from column VGP-2020 of “Table 1: Proposed standards and metrics for defining genome assembly quality” from ( Rhie *et al.*, 2021). ^**^BUSCO scores based on the lepidoptera_odb10 BUSCO set using v5.3.2. C = complete [S = single copy, D = duplicated], F = fragmented, M = missing, n = number of orthologues in comparison. A full set of BUSCO scores is available at https://blobtoolkit.genomehubs.org/view/ilAgrMarg1.1/dataset/CAKOAS01/busco.

**Figure 3.**
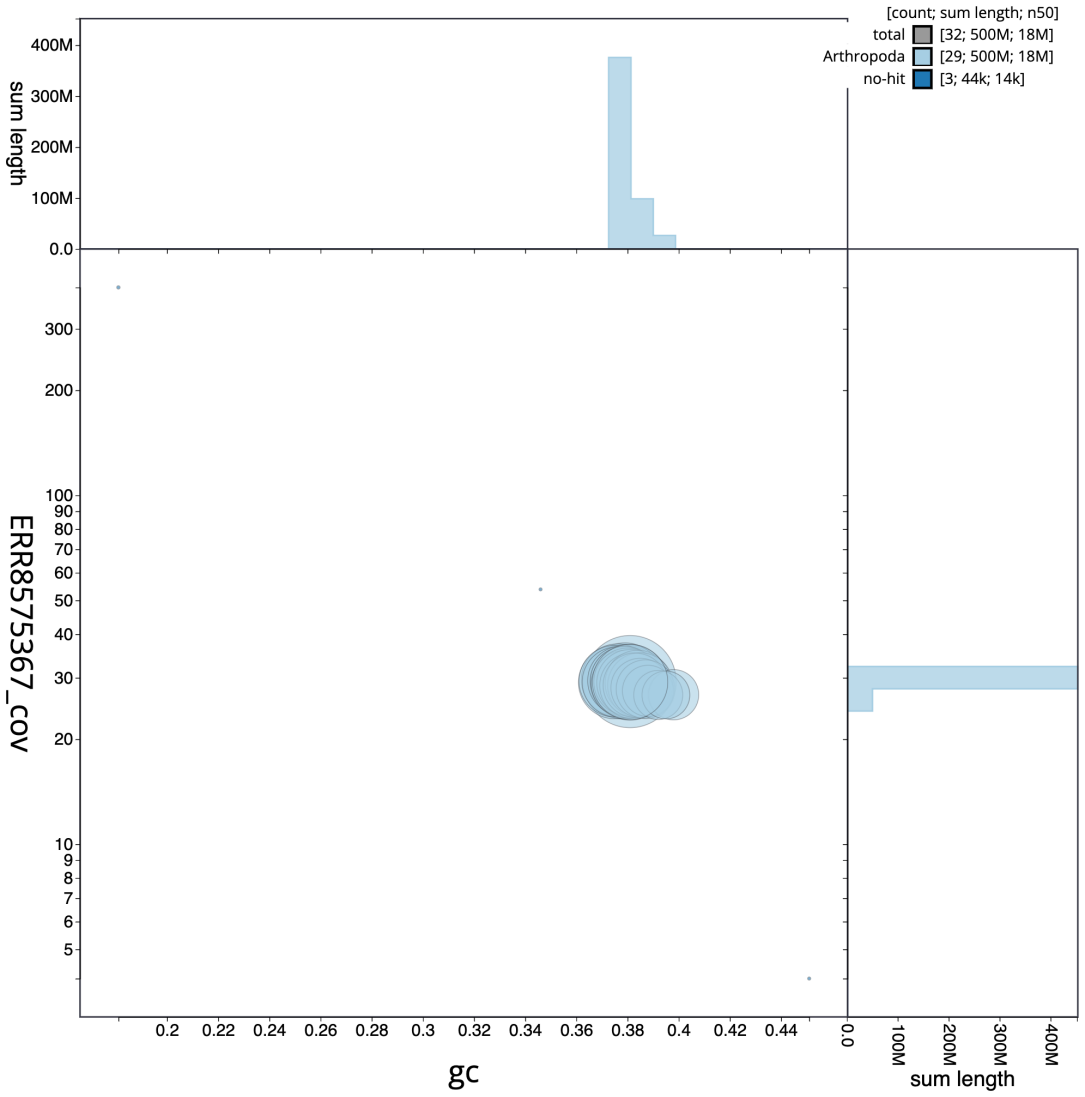
Genome assembly of *Agriopis marginaria*, ilAgrMarg1.1: GC coverage. BlobToolKit GC-coverage plot. Scaffolds are coloured by phylum. Circles are sized in proportion to scaffold length. Histograms show the distribution of scaffold length sum along each axis. An interactive version of this figure is available at https://blobtoolkit.genomehubs.org/view/ilAgrMarg1.1/dataset/CAKOAS01/blob.

**Figure 4.**
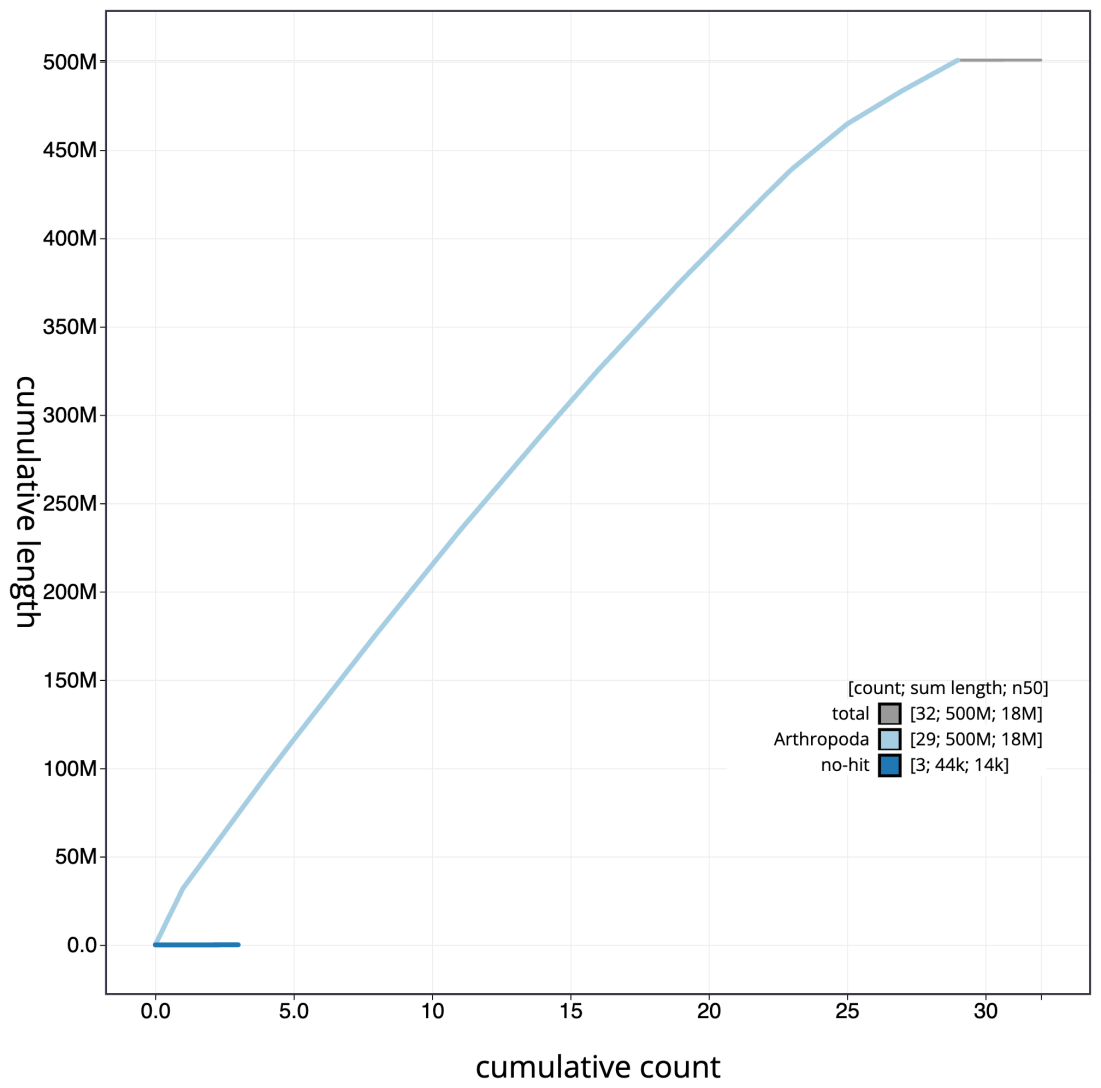
Genome assembly of *Agriopis marginaria*, ilAgrMarg1.1: cumulative sequence. BlobToolKit cumulative sequence plot. The grey line shows cumulative length for all scaffolds. Coloured lines show cumulative lengths of scaffolds assigned to each phylum using the buscogenes taxrule. An interactive version of this figure is available at https://blobtoolkit.genomehubs.org/view/ilAgrMarg1.1/dataset/CAKOAS01/cumulative.

**Figure 5.**
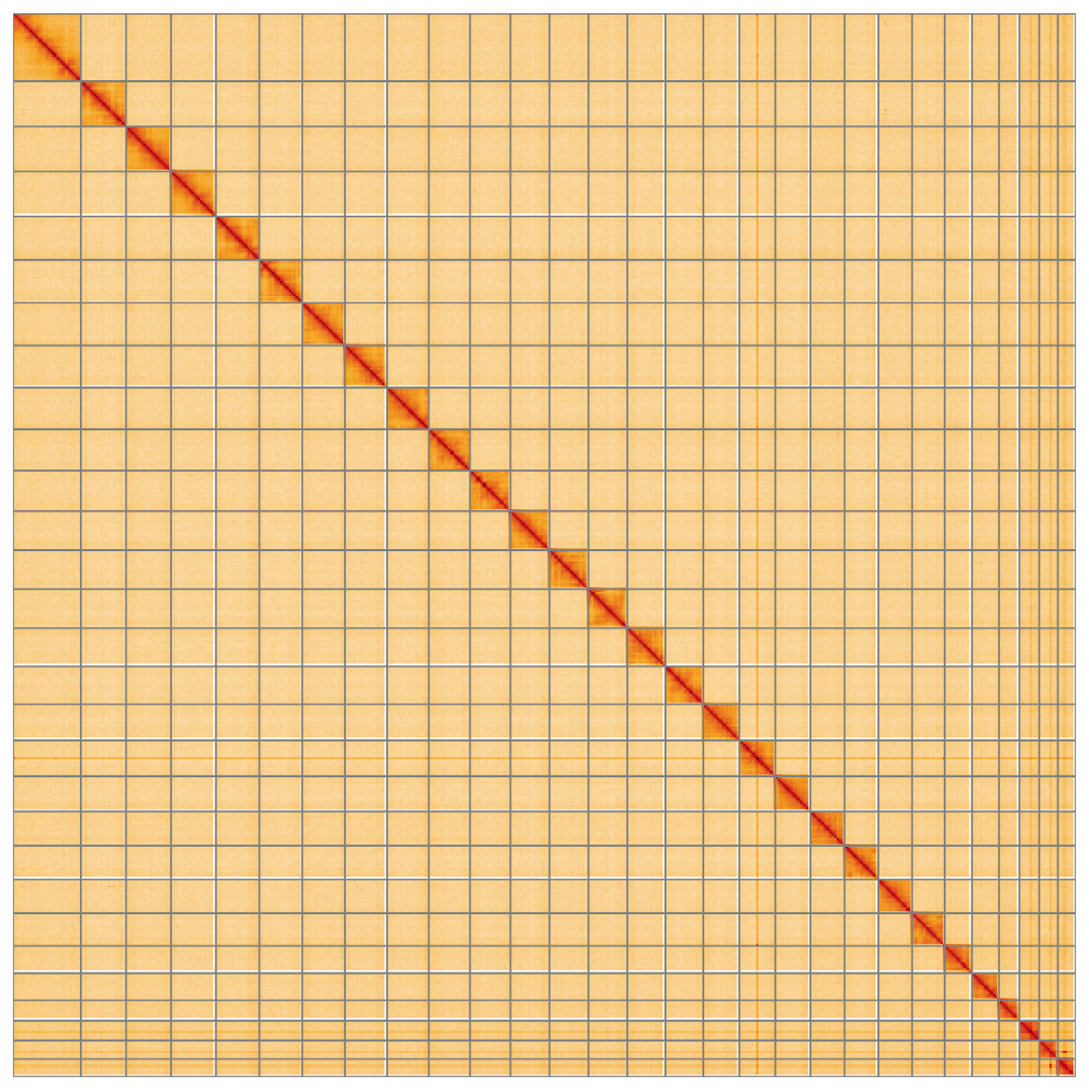
Genome assembly of *Agriopis marginaria*, ilAgrMarg1.1: Hi-C contact map. Hi-C contact map of the ilAgrMarg1.1 assembly, visualised using HiGlass. Chromosomes are shown in order of size from left to right and top to bottom. An interactive version of this figure may be viewed at https://genome-note-higlass.tol.sanger.ac.uk/l/?d=J9_K3bZbSv-I6t4HZGMRhg.

**Table 2.** Chromosomal pseudomolecules in the genome assembly of *Agriopis marginaria*, ilAgrMarg1.

INSDC accession	Chromosome	Size (Mb)	GC%
OW029914.1	1	31.95	38.1
OW029915.1	2	21.32	38.1
OW029917.1	3	21.1	37.9
OW029918.1	4	20.5	37.9
OW029919.1	5	20.24	38
OW029920.1	6	20.09	37.8
OW029921.1	7	19.84	37.7
OW029922.1	8	19.68	37.5
OW029923.1	9	19.38	37.6
OW029924.1	10	19.1	37.5
OW029925.1	11	18.4	37.9
OW029926.1	12	18.36	37.6
OW029927.1	13	18.34	37.9
OW029928.1	14	17.97	37.8
OW029929.1	15	17.85	37.9
OW029930.1	16	17	37.9
OW029931.1	17	16.94	38
OW029932.1	18	16.54	38.1
OW029933.1	19	16.12	38.2
OW029934.1	20	15.93	38.4
OW029935.1	21	15.9	38.5
OW029936.1	22	15.35	38.3
OW029937.1	23	12.88	38.5
OW029938.1	24	12.72	38.7
OW029939.1	25	9.64	38.8
OW029940.1	26	9.28	39.8
OW029941.1	27	8.83	39.2
OW029942.1	28	8.38	39.5
OW029916.1	Z	21.25	38.1
OW029943.1	MT	0.02	18.1

The estimated Quality Value (QV) of the final assembly is 64.6 with *k*-mer based completeness of 100%, and the assembly has a BUSCO v5.3.2 ( Manni *et al.*, 2021) completeness of 98.3% (single 97.8%, duplicated 0.5%) using the lepidoptera_odb10 reference set ( *n* = 5,286).

## Genome annotation report

The *Agriopis marginaria* genome assembly (GCA_932305915.1) was annotated using the Ensembl rapid annotation pipeline ( Table 1; Accession Number: GCA_932305915.1). The resulting annotation includes 21,487 transcribed mRNAs from 12,443 protein-coding and 1,975 non-coding genes.

## Methods

### Sample acquisition and nucleic acid extraction

A male *A. marginaria* specimen (ilAgrMarg1) was collected from High Wycombe, Buckinghamshire, UK (latitude 51.63, longitude –0.74) on 24 February 2021. The specimen was caught by David Lees (Natural History Museum) using a light trap. The specimen was identified by the collector based on morphology and dry frozen at –80°C. The ilArgMarg1 specimen was used for DNA sequencing and Hi-C scaffolding.

A second *A. marginaria* specimen (ilAgrMarg2) was collected by Douglas Boyes (University of Oxford) from Wytham Woods, Oxfordshire (biological vice-county: Berkshire), UK (latitude 51.77, longitude –1.34) on 31 March 2021 using a light trap. The specimen was identified by the collector and snap-frozen on dry ice. This specimen was used for RNA sequencing.

DNA was extracted at the Tree of Life laboratory, Wellcome Sanger Institute (WSI). The ilAgrMarg1 sample was weighed and dissected on dry ice with tissue set aside for Hi-C sequencing. Abdomen tissue was cryogenically disrupted to a fine powder using a Covaris cryoPREP Automated Dry Pulveriser, receiving multiple impacts. High molecular weight (HMW) DNA was extracted using the Qiagen MagAttract HMW DNA extraction kit. HMW DNA was sheared into an average fragment size of 12–20 kb in a Megaruptor 3 system with speed setting 30. Sheared DNA was purified by solid-phase reversible immobilisation using AMPure PB beads with a 1.8X ratio of beads to sample to remove the shorter fragments and concentrate the DNA sample. The concentration of the sheared and purified DNA was assessed using a Nanodrop spectrophotometer and Qubit Fluorometer and Qubit dsDNA High Sensitivity Assay kit. Fragment size distribution was evaluated by running the sample on the FemtoPulse system.

RNA was extracted from abdomen tissue of ilAgrMarg2 in the Tree of Life Laboratory at the WSI using TRIzol, according to the manufacturer’s instructions. RNA was then eluted in 50 μL RNAse-free water and its concentration assessed using a Nanodrop spectrophotometer and Qubit Fluorometer using the Qubit RNA Broad-Range (BR) Assay kit. Analysis of the integrity of the RNA was done using Agilent RNA 6000 Pico Kit and Eukaryotic Total RNA assay.

### Sequencing

Pacific Biosciences HiFi circular consensus DNA sequencing libraries were constructed according to the manufacturers’ instructions. Poly(A) RNA-Seq libraries were constructed using the NEB Ultra II RNA Library Prep kit. DNA and RNA sequencing was performed by the Scientific Operations core at the WSI on the Pacific Biosciences SEQUEL II (HiFi) and Illumina NovaSeq 6000 (RNA-Seq) instruments. Hi-C data were also generated from head and thorax tissue of ilAgrMarg1 using the Arima v2 kit and sequenced on the Illumina NovaSeq 6000 instrument.

### Genome assembly, curation and evaluation

Assembly was carried out with Hifiasm ( Cheng *et al.*, 2021) and haplotypic duplication was identified and removed with purge_dups ( Guan *et al.*, 2020). The assembly was scaffolded with Hi-C data ( Rao *et al.*, 2014) using YaHS ( Zhou *et al.*, 2023). The assembly was checked for contamination as described previously ( Howe *et al.*, 2021). Manual curation was performed using HiGlass ( Kerpedjiev *et al.*, 2018) and Pretext ( Harry, 2022). The mitochondrial genome was assembled using MitoHiFi ( Uliano-Silva *et al.*, 2022), which performed annotation using MitoFinder ( Allio *et al.*, 2020).

To evaluate the assembly, MerquryFK was used to estimate consensus quality (QV) scores and *k*-mer completeness ( Rhie *et al.*, 2020). The genome was analysed, and BUSCO scores ( Manni *et al.*, 2021; Simão *et al.*, 2015;) were generated within the BlobToolKit environment ( Challis *et al.*, 2020). Table 3 contains a list of software tool versions and sources.

**Table 3.** Software tools: versions and sources.

Software tool	Version	Source
BlobToolKit	4.0.7	https://github.com/blobtoolkit/ blobtoolkit
BUSCO	5.3.2	https://gitlab.com/ezlab/busco
Hifiasm	0.16.1-r375	https://github.com/chhylp123/ hifiasm
HiGlass	1.11.6	https://github.com/higlass/higlass
Merqury	MerquryFK	https://github.com/thegenemyers/ MERQURY.FK
MitoHiFi	2	https://github.com/marcelauliano/ MitoHiFi
PretextView	0.2	https://github.com/wtsi-hpag/ PretextView
purge_dups	1.2.3	https://github.com/dfguan/purge_ dups
YaHS	yahs- 1.1.91eebc2	https://github.com/c-zhou/yahs

### Genome annotation

The Ensembl gene annotation system ( Aken *et al.*, 2016) was used to generate annotation for the *Agriopis marginaria* assembly (GCA_932305915.1). Annotation was created primarily through alignment of transcriptomic data to the genome, with gap filling via protein-to-genome alignments of a select set of proteins from UniProt ( UniProt Consortium, 2019).

### Ethics and compliance issues

The materials that have contributed to this genome note have been supplied by a Darwin Tree of Life Partner. The submission of materials by a Darwin Tree of Life Partner is subject to the Darwin Tree of Life Project Sampling Code of Practice. By agreeing with and signing up to the Sampling Code of Practice, the Darwin Tree of Life Partner agrees they will meet the legal and ethical requirements and standards set out within this document in respect of all samples acquired for, and supplied to, the Darwin Tree of Life Project. All efforts are undertaken to minimise the suffering of animals used for sequencing. Each transfer of samples is further undertaken according to a Research Collaboration Agreement or Material Transfer Agreement entered into by the Darwin Tree of Life Partner, Genome Research Limited (operating as the Wellcome Sanger Institute), and in some circumstances other Darwin Tree of Life collaborators.

## Data Availability

European Nucleotide Archive: *Agriopis marginaria* (Dotted Border). Accession number PRJEB50734; https://identifiers.org/ena.embl/PRJEB50734 ( Wellcome Sanger Institute, 2022) The genome sequence is released openly for reuse. The *Agriopis marginaria* genome sequencing initiative is part of the Darwin Tree of Life (DToL) project. All raw sequence data and the assembly have been deposited in INSDC databases. Raw data and assembly accession identifiers are reported in Table 1.

## Author information

Members of the Natural History Museum Genome Acquisition Lab are listed here: https://doi.org/10.5281/zenodo.4790042.

Members of the University of Oxford and Wytham Woods Genome Acquisition Lab are listed here: https://doi.org/10.5281/zenodo.4789928.

Members of the Darwin Tree of Life Barcoding collective are listed here: https://doi.org/10.5281/zenodo.4893703.

Members of the Wellcome Sanger Institute Tree of Life programme are listed here: https://doi.org/10.5281/zenodo.4783585.

Members of Wellcome Sanger Institute Scientific Operations: DNA Pipelines collective are listed here: https://doi.org/10.5281/zenodo.4790455.

Members of the Tree of Life Core Informatics collective are listed here: https://doi.org/10.5281/zenodo.5013541.

Members of the Darwin Tree of Life Consortium are listed here: https://doi.org/10.5281/zenodo.4783558.
