# Supplementary material for: The genome sequence of the Dotted Border, Agriopis marginaria (Fabricius, 1776)
Source: Wellcome Open Res. 2023 Mar 31;8:152. [Version 1] doi: 10.12688/wellcomeopenres.19284.1 (PMC10435923; doi:10.12688/wellcomeopenres.19284.1)
Supplement: Peer review history

# Peer review: Reviewer response for version 1

**DOI:** 10.21956/wellcomeopenres.21367.r64604

**Published:** 2023-08-21

**Authors:** Francis M.F. Nunes

**Affiliations:** 1 Departamento de Genética e Evolução, Universidade Federal de Sao Carlos, São Carlos, State of São Paulo, Brazil

The authors sequenced and assembled the ~ 501 Mb genome of the dotted border moth, *Agriopis marginaria*.

 Despite being a concise Data Note, the authors could enhance the article's quality and improve the scientific community's understanding by investing further in elaborating the rationale behind dataset creation and incorporating a foundational biological interpretation of the outcomes. For example: Have the authors identified genes that might be associated with (i) larval polyphagy, (ii) adult flightlessness, and (iii) the development of melanism? If feasible, kindly provide some of the discoveries and/or genomic insights derived from sequencing data.

- Do ilAgrMarg1 and ilAgrMarg2 belong to the adult stage? Additionally, is the sex of the second *A. marginaria* specimen (ilAgrMarg2) male or female?
- The approach employed for aligning transcriptomic data to the genome lacks clarity.
- Could the authors please consider adding a scale in millimeters or centimeters to Figure 1? This would provide a sense of the insect's size.
- Figures 2 to 5 possess significance; however, they are only cursorily examined, rendering them less informative for prospective readers of the article. Despite the article's nature as a 'data in brief report', it is crucial to maintain a sufficient level of coherence and cohesion between the text and figures. Please, improve these parts.

Are sufficient details of methods and materials provided to allow replication by others?

Partly

Is the rationale for creating the dataset(s) clearly described?

Yes

Are the datasets clearly presented in a useable and accessible format?

Yes

Are the protocols appropriate and is the work technically sound?

Partly

Reviewer Expertise:

Genetics, genomics, (epi)transcriptomics, gene silencing (RNAi), insects, aging, nutrition, metamorphosis.

I confirm that I have read this submission and believe that I have an appropriate level of expertise to confirm that it is of an acceptable scientific standard, however I have significant reservations, as outlined above.

# Peer review: Reviewer response for version 1

**DOI:** 10.21956/wellcomeopenres.21367.r64601

**Published:** 2023-08-17

**Authors:** Edson Ishengoma

**Affiliations:** 1 University of Dar es Salaam, Dar es Salaam,, Tanzania

The genome of a moth species known as Dotted Border, *Agriopis marginaria*, is reported in this data note for the first time. Because this is a data note, there aren’t many information on how this genome informs the biology and ecology of the species, and the authors believe this may be done later. The basic concept of genome quality, as well as methodologies to genome assembly and annotation, is quite standard. What is likely missing for the time being is the inclusion of distinct developmental phases in the generated gene models of this insect. I’m particularly curious how the 30 chromosomal pseudomolecules, which include the Z and mitochondrial haplotypes, compare to the number of pseudomolecules found in other related moth species.

Are sufficient details of methods and materials provided to allow replication by others?

Yes

Is the rationale for creating the dataset(s) clearly described?

Yes

Are the datasets clearly presented in a useable and accessible format?

Yes

Are the protocols appropriate and is the work technically sound?

Yes

Reviewer Expertise:

Genomics, Bioinformatics, Evolutionary Biology

I confirm that I have read this submission and believe that I have an appropriate level of expertise to confirm that it is of an acceptable scientific standard.

# Peer review: Reviewer response for version 1

**DOI:** 10.21956/wellcomeopenres.21367.r56799

**Published:** 2023-05-19

**Authors:** Francesco Frati, Claudio Cucini

**Affiliations:** 1 University of Siena, Siena, Tuscany, Italy; 2 Life Sciences, University of Siena, Siena, Tuscany, Italy

This work aims to publicly release the genome of *Agriopis marginaria* after an important sequencing effort. The sequencing process and the subsequent data validations (BUSCO, Blobtools, genome annotation) are consistent with the currently available pipelines and gold standards for genome sequencing and annotation.

 Despite only a few (in my opinion) genes are recovered during the annotation step – and this could be improved in further, more detailed analyses (for example by adding transcriptome sequencing of multiple instars) - the work appears solid and provides a lot of data that can be used for further genomic analyses (comparative genomics, phylogenomics, etc.).

 Just a couple of minor issues:

- We suggest selecting keywords that are NOT already present in the title.
- We find the indication of the longitude of the collecting sites a bit weird (-0.74; -1.34). Shouldn't it be used the usual notation East or West (we assume West in this case?)

We recommend the Approval of the article.

Are sufficient details of methods and materials provided to allow replication by others?

Yes

Is the rationale for creating the dataset(s) clearly described?

Yes

Are the datasets clearly presented in a useable and accessible format?

Yes

Are the protocols appropriate and is the work technically sound?

Yes

Reviewer Expertise:

Evolutionary Biology; Entomology; Phylogeny

We confirm that we have read this submission and believe that we have an appropriate level of expertise to confirm that it is of an acceptable scientific standard.
